# Direct cell extraction of membrane proteins for structure–function analysis

**DOI:** 10.1038/s41598-023-28455-w

**Published:** 2023-01-25

**Authors:** Ieva Drulyte, Aspen Rene Gutgsell, Pilar Lloris-Garcerá, Michael Liss, Stefan Geschwindner, Mazdak Radjainia, Jens Frauenfeld, Robin Löving

**Affiliations:** 1 Thermo Fisher Scientific, Materials and Structural Analysis, Achtseweg Noord 5, 5651 GG Eindhoven, The Netherlands; 2 AstraZeneca, Mechanistic and Structural Biology, Discovery Sciences, R&D, 431 50 Mölndal, Sweden; 3 Salipro Biotech, Teknikringen 38A, 114 28 Stockholm, Sweden; 4 Thermo Fisher Scientific GENEART GmbH, Im Gewerbepark B35, 93059 Regensburg, Germany

**Keywords:** Biological techniques, Biophysics, Drug discovery, Structural biology

## Abstract

Membrane proteins are the largest group of therapeutic targets in a variety of disease areas and yet, they remain particularly difficult to investigate. We have developed a novel one-step approach for the incorporation of membrane proteins directly from cells into lipid Salipro nanoparticles. Here, with the pannexin1 channel as a case study, we demonstrate the applicability of this method for structure–function analysis using SPR and cryo-EM.

## Introduction

Membrane proteins are central to many physiological processes and are of critical importance for the pharmaceutical industry. More than 60% of all current clinically-approved drugs target proteins embedded in the lipid membrane^1,2^. However, membrane proteins are particularly difficult to investigate in drug discovery campaigns because they are notoriously difficult to purify using standard purification methods.

To address this problem, the Salipro nano-membrane system was developed as a universal platform to stabilize membrane proteins in a native-like lipid environment using a scaffold of saposin proteins^3–6^. The Salipro platform has recently expanded to enable the direct extraction of sensitive membrane proteins from crude cell membranes, thereby eliminating the need for detergent-purification^7^. This methodology is termed DirectMX for direct membrane extraction. In this work, we present a case study using a streamlined version of the DirectMX protocol working directly from crude cell pellets to purify the ATP release channel pannexin1 (PANX1), and investigate its structural and ligand-binding properties.

PANX1 is a potential therapeutic target and associated with various pathologies, including inflammation, pain, ischemia, and epilepsy^8,9^. A major limitation for exploring PANX1 biology, or its potential as a therapeutic target, is an overall lack of biophysical studies using isolated systems. To date, several pharmacological agents have been reported to inhibit PANX1 using cellular functional assays; however, it is impossible to distinguish if the observed phenotypic effects are directly from PANX1 target engagement or due to indirect effects from binding to other proteins^10,11^. Here, we aimed to use the Salipro DirectMX method, working directly from cell pellets, to isolate PANX1 and perform the first-ever in vitro structure–function assays using surface plasmon resonance (SPR) and single-particle cryo electron microscopy (cryo-EM).

## Results

**Figure 1:**
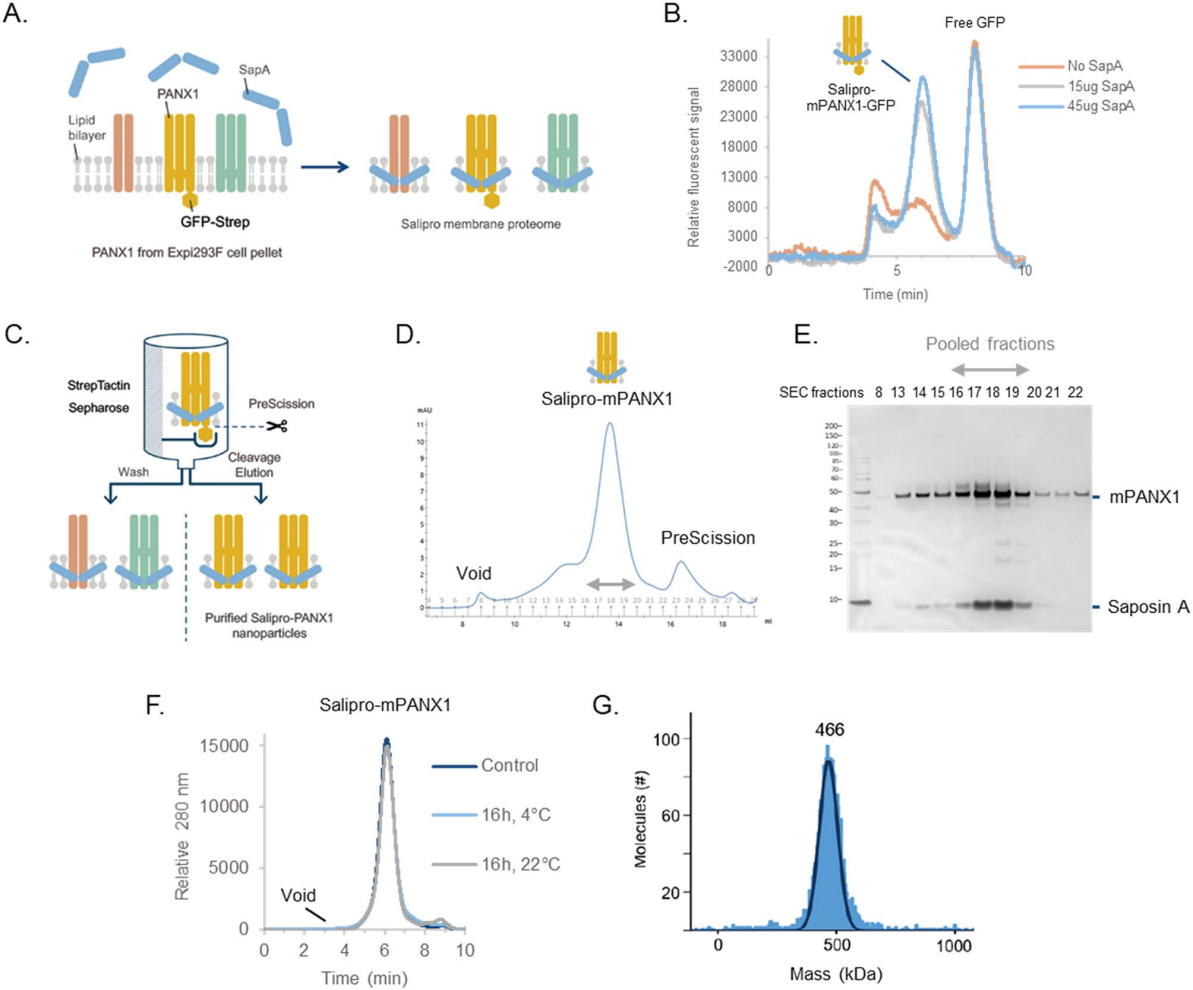
Salipro-mPANX1 DirectMX summary. (**A**) Schematic illustration of the DirectMX methodology. (**B**) mPANX1 expressed as a GFP fusion protein formed a homogenous population of Salipro-mPANX1-GFP nanoparticles upon addition of SapA, peaking around 6 min. (**C**) Schematic illustration of the Salipro-mPANX1 affinity purification using StrepTactin Sepharose with on-column PreScission cleavage. (**D**) Salipro-mPANX1 particle purification by preparative SEC. (**E**) SEC fraction analysis by SDS-PAGE. The unprocessed and uncropped SDS-PAGE image is shown in Supplementary Fig. S1. (**F**) Analytic SEC trace of Salipro-mPANX1 particles that were thawed and incubated for 16 h at 4 °C or 22 °C. The control sample was analyzed immediately after freeze-thawing without any further incubation. (**G**) Mass photometry confirms the presence of a homogenous population of Salipro-mPANX1 particles with an apparent nanoparticle size of 466 kDa.

To begin, expression constructs for mouse PANX1 (mPANX1) containing cleavable C-terminal GFP and TwinStep affinity tags were transiently expressed in Expi293 cells. Cell pellets were harvested 5 days post-transfection, and stored at − 80 °C. Cell pellets were resuspended in digitonin-containing buffer to increase membrane fluidity and render lipids and membrane proteins more accessible for reconstitution using saposin A (Fig. 1A). Following a 10-min incubation with saposin A, the formation of saposin-containing mPANX1-GFP particles was assessed by analytical size exclusion chromatography (SEC) using a fluorescens detector (Fig. 1B). For affinity purification, affinity-tagged mPANX1-containing particles were immobilised on a StrepTactin Sepharose column and eluted using the PreScission protease for on-column cleavage (Fig. 1C). Next, the eluate was concentrated by ultrafiltration and subjected to preparative SEC (Fig. 1D). SEC fractions were analyzed by silver staining after SDS-PAGE revealed the formation of pure and homogenous Salipro-mPANX1 nanoparticles (Fig. 1E). SEC fractions containing Salipro-mPANX1 were pooled, concentrated and flash frozen prior to use in downstream applications.

Structure–function analysis of membrane proteins require a stable sample and we therefore assessed the thermal stability of freeze-thawed Salipro-mPANX1 particles at 4 °C or 22 °C for 16 h using analytic SEC (Fig. 1F). Salipro-mPANX1 particles remained stable and homogenous at 22 °C for up to 16 h (Fig. 1F). Mass photometry measurements^12^ also showed a homogenous population of freeze-thawed Salipro-mPANX1 particles at a molecular size of approximately 466 kDa (Fig. 1G). Analogous to the DirectMX extraction of mPANX1, we applied the same methodology for human PANX1 (hPANX1) expressed in sf9 insect cells, leading to similar results (Supplementary Fig. S2).

**Figure 2:**
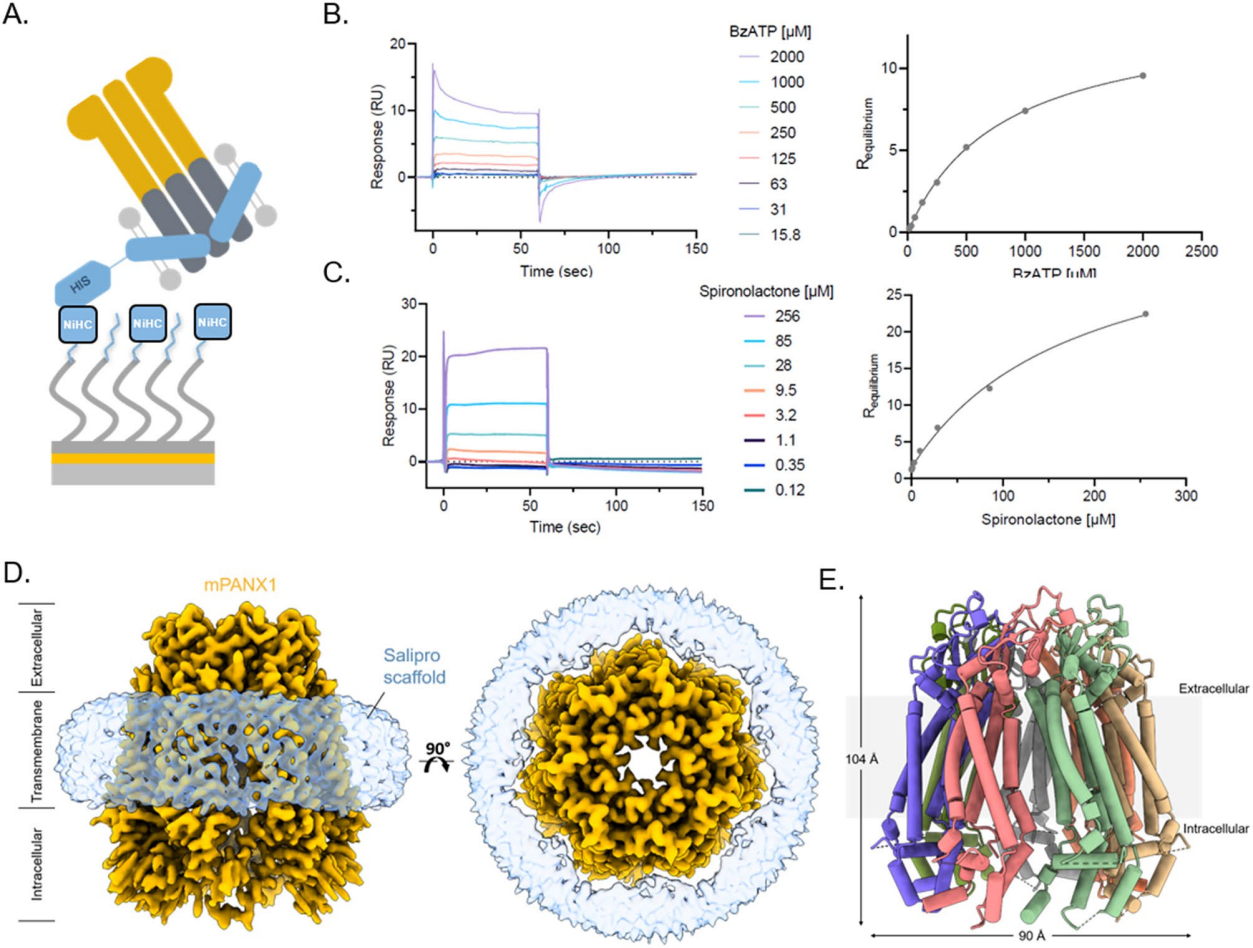
Salipro-mPANX1 structure and functional analysis. (**A**) Schematic of His-Salipro-mPANX1 nanoparticles immobilisation to a sensor with Ni^2+^ ions (NiHC) binding to the His-tag. Concentration series of bzATP (**B**) and spironolactone (**C**) was injected over a His-Salipro-mPANX1 coated surface with a calculated K_D_ of 720 μM ± 133 μM and 160 μM ± 10 μM, respectively. (**D**) Cryo-EM reconstruction of Salipro-mPANX1 at 3.1 Å. The ion channel (orange) and Salipro lipid disk (blue) is shown as side view (left) and top view (right). (**E**) The structural model of mPANX1 with each subunit colored. All SPR data is representative of n = 3.

We then employed SPR to directly assess the ligand-binding competence of purified Salipro-PANX1 particles. In the case of membrane proteins, SPR analysis remains challenging due to the intrinsic instability of native membrane proteins solubilized in detergents and the difficulty to immobilize enough functional membrane proteins to obtain significant binding signal intensity when working with small molecular ligands^13^. Untagged mPANX1 embedded in a His-tagged Salipro nanoparticle was immobilized (Fig. 2A) and challenged with benzoylbenzoyl-ATP (bzATP), spironolactone, and carbenoxolone representing known inhibitors with previously reported IC50 inhibition of PANX1 voltage stimulation values^10^. In vitro binding constants were determined for bzATP (K_D_ 720 μM ± 133 μM) and spironolactone (K_D_ 160 μM ± 10 μM) (Fig. 2B,C). Binding to carbenoxolone was also confirmed, although the binding constant could not be determined most likely because higher carbenoxolone concentrations could not be explored due to limitations in compound solubility (Supplementary Fig. S3). Similar results were observed for biotin-Salipro-hPANX1 nanoparticles (Supplementary Fig. S4) with all SPR data summarized in Supplementary Table S1. In summary, the downstream analysis showed a high degree of functional Salipro-PANX1 material immobilized on the sensor surface with an approximate binding capacity to small molecular compounds between 80 and 90%.

Lastly, we present the first cryo-EM structure of a membrane protein that was directly reconstituted from crude cell pellets into Salipro nanoparticles (Fig. 2D,E). To date, all cryo-EM structures of membrane proteins in Salipro particles were obtained from membrane proteins that were detergent-purified first and, in a second step, reconstituted with an artificial mix of lipids^3–6^. Briefly, cryo-EM grids were prepared using Salipro-mPANX1 nanoparticles and standard Vitrobot plunge-freezing. Cryo-EM micrographs revealed that frozen-hydrated complexes were monodisperse with 2D class averages displaying secondary and tertiary structures, as well as heptameric organization of mPANX1 (Supplementary Fig. S5). Using a total of 269,000 particles, the 3D structure reconstruction reached a 3.1 Å resolution (Supplementary Fig. S5 and Supplementary Table S2).

Extracellular and transmembrane regions of the Salipro-mPANX1 are well-resolved in the structure with clear side-chain density visible (Supplementary Figs. S6A and S7). In contrast, much of the intracellular regions were not well resolved in the cryo-EM map prohibiting complete atomic modelling. To examine the quality of the structure map, 2D class averages (Supplementary Fig. S5D) showed multiple tilted views and the corresponding 3DFSC analysis^14^ shown in Supplementary Fig. S8, reports the sphericity of the map to be 0.92 out of 1, showing very little directional anisotropy and suggesting that the intracellular region of the map displays the lowest resolution due to its inherent flexibility as opposed to any limitation in data quality. We next compared Salipro-mPANX1 to hPANX1 (PDB code 6WBF), a homologue with a shared sequence identity of 86.2%. The dimensions of the Salipro-mPANX1 heptamer are 90 by 104 Å (Fig. 2E), which is slightly smaller than those of hPANX1, which are 96 by 109 Å. On the protomer level, the mouse and human PANX1 structures are highly similar, with a root-mean-square deviation (RMSD) of 1.556 across 256 aligned atom pairs. The most striking difference between the two structures can be observed in the N-terminus, where hPANX1 reveals a structured N-terminal alpha helix (NTH) in the channel pore^15^. On the other hand, the N-terminus of Salipro-mPANX1 could not be resolved (Supplementary Fig. S6C), similar to what has been reported for several other PANX1 structures^16–20^. Furthermore, the structured intracellular region of Salipro-mPANX1 exhibits some minor but clear differences compared to the hPANX1 with several alpha-helices shifted; for example, helices encompassing residues 342–349 (Salipro-mPANX1) and 342–355 (hPANX1) reach a maximum displacement of ~ 5 Å (Supplementary Fig. S6B,C).

## Discussion

In this proof-of-concept study, we present the Salipro method for the reconstitution and purification of homogenous wildtype mPANX1 and hPANX1 particles directly from cell pellets. Further, we developed a PANX1 in vitro small molecular ligand-binding assay, enabling screening compounds for this drug target via SPR. The cryo-EM structure of mouse PANX1 was solved (3.1 Å), representing the first published structure of a membrane protein extracted by Salipro nanoparticles directly from cells.

Several PANX1 structures have previously been solved and all of them approximately show similar overall structures albeit with differences in the intracellular domains^15–22^ and lipid-like densities at the membrane interface^15,16,22^. Overall, our Salipro-mPANX1 structure is in agreement with several published structures of hPANX1 that display poorly resolved N-termini, suggesting there is flexibility in the intracellular space. This flexibility has been hypothesized to be involved in the formation of a channel gate with the N-terminus having potential roles in different gating mechanisms^16–20^. With the current setup, we could not resolve the N-terminus of the Salipro-mPANX1 structure, so we cannot directly comment on the functional role of this region. In addition, it was not possible to resolve any lipid-like density in the Salipro-mPANX1 structure. Since our structure contains endogenous lipids from mPANX1-expressing cells, this lack of lipid-like density could suggest that endogenous lipids have a certain flexibility surrounding mPANX1. Other PANX1 structures have resolved lipid-density in the transmembrane domain but this difference is likely due to differences in purification methods^15,16,22^.

A variety of pharmacological agents have been reported to inhibit PANX1 channels using cellular patch-clamping experiments^10^. Until now, it has not been possible to determine if the majority of these compounds effect PANX1 function through direct binding. Here, we were able to develop a direct-binding assay for Salipro-PANX1 using SPR. Overall, we found that there is quite a substantial difference between reported IC50 values and the KD values determined with SPR. For example, spironolactone and carbenoxolone have reported IC50 values of 7 µM and 5 µM^10^, respectively whereas we found the affinity to be significantly weaker for both compounds with a KD of 160 µM for spironolactone and a KD > 256 µM for carbenoxolone using SPR. A possible explanation to this dramatic drop in potency may be related to the specificity of these compounds. Carbenoxolone and spironolactone are known to interact with other membrane proteins. Therefore, it is possible that these compounds have a more complicated effect on PANX1 by interacting with other membrane proteins rather than through a direct-binding mechanism to PANX1 itself.

Ions such as K^+^ and Ca^2+^ are known to functionally affect the PANX1 channel^10,11^ and mechanistic explanations for these observation remain to be resolved^15^. Although outside the scope of this manuscript, it would be interesting to conduct SPR and cryo-EM experiments with Salipro-PANX1 in the presence of K^+^ and Ca^2+^ to explore if these ions affect the PANX1 structure and its binding to inhibitors. In addition, investigations of novel PANX1-binders using the combination of SPR and cryo-EM could lead to the development of more potent and specific compounds to further probe the biology of PANX1 and its potential as therapeutic target.

Overall, the direct cell extraction presented here enables purification of stable and functional wildtype membrane proteins without the need for laborious and time-consuming detergent screenings, protein engineering or screening of applicable alternative scaffolding setups. We believe that the Salipro direct cell extraction may accelerate future membrane protein research, as well as enable the development of new therapeutic ligands in a time-efficient and streamlined manner.

## Methods

### Material and reagents

Human saposin A was recombinantly produced and purified as previously described^3,6^. Pure saposin A in HNG buffer (50 mM HEPES at pH 7.5, 150 mM NaCl, 5% (v/v) glycerol) was stored at − 80 °C. saposin A empty nanoparticles (Salipro-Empty particles) were prepared as previously described^3^. PreScission protease was purchased from Cytiva. PANX1 ligands for SPR binding measurements were Benzoylbenzoyl-ATP (Sigma-Aldrich, CAS 112898-15-4), Carbenoxolone (sourced at AstraZeneca) and Spironolactone (sourced at AstraZeneca).

### PANX1 expression

Two PANX1 expression constructs were designed to express mPANX1 and hPANX1. The mPANX1 construct was designed into the pcDNA3.4 TOPO™ expression vector (Thermo Fisher Scientific) to contain the wildtype mouse PANX1 sequence with a N-terminal FLAG tag and a C-terminal GFP fused to a Twin Strep tag. In addition, the mPANX1 sequence was designed to be flanked by two PreScission protease cleavage sites (FLAG-3C-mPANX1-3C-GFP-StrepII) enabling purification of mPANX1 without the presence of GFP and tags in the final preparation. The expression construct was codon-optimized for protein expression in human cells. Human Expi293 cells were transiently transfected, and cell pellets were collected 5 days post transfection (GeneArt, Thermo Fisher Scientific).

The hPANX1 construct was designed into the pFastBac1 expression vector to contain the wildtype human PANX1 sequence with a C-terminal eGFP fused to a His10 tag followed by an EPEA affinity tag. In addition, a PreScission protease cleavage site was designed directly downstream of hPANX1 (hPANX1-3C-GFP-His10-EPEA) enabling purification of hPANX1 without the presence of GFP and tags in the final preparation. The expression construct was codon optimised for protein expression in insect cells. Viral stocks were prepared and used to induce protein expression in sf9 insect cells. Cell pellets of hPANX1 expressing cells were collected 48 h post infection (GeneArt, Thermo Fisher Scientific). All cell pellets expressing PANX1 were frozen and stored at − 80 °C.

### Membrane protein reconstitution screening using fluorescence-detection size-exclusion chromatography (FSEC)

Cell pellet expressing mPANX1-GFP was thawed and resuspended with a five-time excess of HNG buffer with 1.2 × cOmplete protease inhibitor cocktail (Roche) and 1.2% digitonin (Calbiochem). The mixtures were then incubated for 60 min on a rotating wheel, followed by centrifugation at 30,000*g* for 45 min to remove insoluble material. The sample was then divided into three mixtures (5 μl each) supplemented either with 45 μl 0.3 mg/ml or 1.0 mg/ml saposin A followed by a 10 min incubation. The third sample was occluded from saposin A, serving as negative control. All steps were performed at 4 °C. The formation of saposin A reconstituted mPANX-GFP nanoparticles (Salipro-mPANX1-GFP) was evaluated by analytic size exclusion chromatography (SEC) using a fluorescent detector (FSEC) in a Superose 6 Increase 5/150 GL column (Cytiva) equilibrated with detergent free HNG buffer, using a Prominence-i LC-2030C high performance liquid chromatography system equipped with PDA and RF-20Axs fluorescence detectors (Shimadzu) at a flow rate of 0.3 mL/min. In-line light absorbances were monitored for total protein elution at 280 nm and for GFP elution at 512 nm, with excitation for the latter set to 500 nm.

### Salipro-PANX1 particle purification and characterization

For affinity chromatography to purify Salipro-mPANX1, 500 µL of Strep Tactin Sepharose High Performance resin (GE Healthcare) were equilibrated with HNG buffer in a Poly-prep® Chromatography column (Bio-Rad). The lysate mixture described above (originating from 2.5-g cell pellet) was loaded to the column and the sample flow-through was passed 3 more times over the resin. The PANX1-loaded affinity resin was then incubated with 45 ml HNG buffer supplemented with 1 mg/ml saposin A and incubated for 30 min at 4 °C before discarding the flow-through. After washing the affinity resin with 16 CV HNG buffer, the beads with bound Salipro-PANX1-GFP were resuspended with 600 µL HNG buffer containing 60U of PreScission protease (Cytiva) and incubated at 4 °C overnight in a rotating wheel. After cleavage, the mixture was loaded back into the empty Poly-prep® column and purified Salipro-mPANX1 particles were eluted in the flow-through using 3 CV HNG buffer, while the GFP-tag remained bound to the resin. The elution was concentrated with Amicon filter device with 50-kDa NMWL (Millipore) and subjected to preparative SEC in a Superose 6 Increase 10/300 GL column (Cytiva), equilibrated in HNG buffer, using an Äkta Pure chromatography system (Cytiva) at a flow rate of 0.35 mL/min. The fractions were collected and analysed by SDS-PAGE using NuPAGE 4–12% Bis–Tris polyacrylamide gels (Thermo Fisher Scientific) and visualized by Silver Stain. Fractions containing pure Salipro-mPANX1 nanoparticles were pooled, concentrated as described above, flash frozen and stored at − 80 °C.

Salipro-hPANX1 particles were made using 1-g thawed hPANX1 expressing sf9 cell pellet resuspended with a nine-time excess of HNG buffer with 1.1 × cOmplete protease inhibitor cocktail (Roche) and 1% DDM. The cell mixture was incubated for 60 min on a rotating wheel, followed by centrifugation at 30,000*g* for 45 min to remove insoluble material. Cleared sample was loaded to a 300 μl eCaptureSelect™ C-tag Affinity Matrix (Thermo Fisher Scientific) column and the sample flow-through was passed 3 more times over the resin. The hPANX1-loaded affinity resin was then incubated with 24 ml HNG buffer supplemented with 1 mg/ml saposin A and incubated for 30 min at 4 °C before discarding the flow-through. After washing the affinity resin with 25 CV HNG buffer, the beads with bound Salipro-hPANX1 were resuspended with 400 µL HNG buffer containing 60U of PreScission protease (Cytiva) and incubated at 4 °C overnight in a rotating wheel. After cleavage, the mixture was loaded back into the empty Poly-prep® column and purified Salipro-hPANX1 particles were eluted in the flow-through using 3 CV HNG buffer, while the GFP-tag remained bound to the resin. Salipro-hPANX1 concentration, SEC preparation and SEC fraction analysis were performed as described above.

To evaluate particle stability, purified Salipro-mPANX1 and Salipro-hPANX1 particles were thawed after flash freezing and incubated for 16 h at either 4 °C or 22 °C prior to analytic SEC.

### Saposin A labelled Salipro-PANX1 particles

To generate His-tagged or biotinylated Salipro-PANX1 particles, the same method as described above was used to extract and purify Salipro-PANX1 using His-tagged or biotinylated saposin A. His-tagged saposin A was produced and purified as previously described^3,6^, albeit without the TEV protease cleavage step for His-tag removal. Saposin A was biotinylated in a one-hour incubation step at RT using NHS-PEG12-Biotin (Thermo Fisher Scientific), following the manufacturer’s recommendations. The Quant*Tag™ Biotin Kit (Vector Laboratories) was used to confirm the amount of saposin A biotinylation.

### SPR analysis

All SPR experiments were conducted on a BIAcore S200 or T200 instrument (Cytiva) using 10 mM HEPES pH 7.4, 150 mM NaCl (HBS-N) as the running buffer. Estimated binding capacity was calculated as follows:

$${Theoretical \,R}_{max} = \frac{{R}_{Ligand}\times {MW}_{analyte}\times Valency}{{MW}_{ligand}}$$

where R_ligand_ is the response (RU) of immobilized ligand. Then, the binding capacity can be estimated using the following formula:

$$Binding \,Capacity = \frac{{Experimental \,R}_{max}}{Theoretical {\,R}_{max}}\times 100 (\%)$$

where the experimental R_max_ is the maximum response observed upon analyte binding in saturating conditions.

#### Salipro-mPANX1 SPR assays

Multicycle kinetic experiments were conducted using a sensor with Ni^2+^ ions complexed on a two-dimensional chelating surface (NiHC 1500 M, Xantec Bioanalytics GmbH) at 12 °C. Prior to ligand immobilization, the sensor was washed with 300 mM EDTA pH 8.3 and loaded with Ni^2+^-ions by injecting a 500 nM solution of NiCl2 in running buffer for 3 min. His-tagged saposin A empty nanoparticles (His-Salipro-empty) and his-tagged saposin A mPANX1 particles (His-Salipro-mPANX1) were injected at a concentration of 10–20 ng/mL using a contact time of 16 min at 1μL/min to achieve desired densities of 2500–4000 RU. His-Salipro-Empty were immobilized on the reference surface as a lipid-only control, serving as a control for nonspecific binding. Increasing concentrations of (0.12, 0.35, 1.1, 3.2, 9.5, 28, 85, and 265 μM) of carbenoxolone or spironolactone or (0.03, 0.06, 0.13, 0.25, 0.50, 1.00, 2.00, 4.00 mM) of benzoylbenzoyl-ATP were iteratively injected with a 60 s contact time, followed by a 90 s dissociation phase. All resulting sensorgrams were reference and blank subtracted prior to fitting.

#### Biotin-Salipro-hPANX1 SPR assays

Multicycle kinetic experiments were conducted using a streptavidin-coated sensor (Cytiva) at 12 °C. Prior to ligand immobilization, the sensor was washed with 50 mM NaOH, 1 M NaCl for 1 min. Biotin-tagged saposin A empty nanoparticles (Biotin-Salipro-Empty) and biotin-tagged saposin A hPANX1 particles (Biotin-Salipro-hPANX1) were injected at a concentration of 10–20 ng/mL using a contact time of 16 min at 1μL/min to achieve desired densities of 2500–4000 RU. Biotin-Salipro-Empty particles were immobilized on the reference surface as a lipid-only control, serving as a control for nonspecific binding. Increasing concentrations of (2, 4, 8, 16, 32, 64, 128, and 265 μM) of carbenoxolone or spironolactone or (0.03, 0.06, 0.13, 0.25, 0.50, 1.00, 2.00, 4.00 mM) of benzoylbenzoyl-ATP were iteratively injected with a 60 s contact time, followed by a 90 s dissociation phase. All resulting sensorgrams were reference and blank subtracted prior to fitting.

### Mass photometry

All samples were measured in 1X HBS-N buffer (10 mM HEPES pH 7.4, 150 mM NaCl) using the Refeyn OneMP mass photometer (Refeyn Ltd.) with a 60 s acquisition time. The resulting histograms were fitted to Gaussian distributions using DiscoverMP (Refeyn Ltd.) to extract peak contrast and relative amount of each peak (n = 3). Contrast-to-mass conversion was achieved by calibration using NativeMark protein ladder (Thermo Fisher Scientific). Three protein species (with specified masses) were fitted to corresponding Gaussian distributions to extract a linear relation between mass and contrast.

### Cryo-EM sample preparation and data collection

Quantifoil 200 mesh 1.2/1/3 Cu grids were glow-discharged using 20 mAmp current for 45 s and charge set to positive (GloQube®, Quorum Technologies). To overcome preferred orientation, a purified full-length Salipro-mPANX1 protein sample was mixed with 0.5 mM Fos-Choline 8 (Anatrace) before the grid freezing. 3 μl of 2.5 mg/ml protein was pipetted onto the grid in Vitrobot Mark IV (Thermo Fisher Scientific) chamber set to 4 °C and 95% humidity. Grids were then blotted for 10 s using a blot force of + 20 and 30 s waiting time before plunge-freezing in liquid ethane.

Data collection was conducted using a 300 kV Thermo Scientific™ Krios G4™ Cryo-Transmission Electron Microscope (Cryo-TEM) equipped with Selectris X Imaging Filter and Falcon 4™ Direct Electron Detector camera operated in Electron-Event representation (EER) mode. Thermo Fisher Scientific EPU 2 software was used to automate the data collection. Aberration-free image shifts (AFIS) and Fringe-free Imaging (FFI) increased the data collection throughput to ~ 360 images/hour. 7995 movie dataset was collected in EFTEM mode using 10 eV slit and a nominal magnification of 165,000×, resulting in a calibrated pixel size of 0.727 Å/px. The exposure time of 4.16 s with a total dose of 40.24 e^−^/Å^2^ was used, and each movie was split into 40 fractions during motion correction. The dose rate on the camera was 5.4 e^−^/px/s, and the nominal defocus range was specified between −0.5 and −1.5 µm in 0.25 µm intervals.

### Cryo-EM single-particle analysis

Data processing was performed using Relion 3^24^ and CryoSPARC™^25^ image processing suites. After motion- and CTF-correction (using Relion's implementation of MOTIONCOR^26^ and CTFFIND-4.1^27^, respectively), particles were picked using a template-free auto-picking procedure based on a Laplacian-of-Gaussian (LoG) filter with particle diameter set to 130–170 Å. A total of 1,314,251 particles were picked from 6739 micrographs and exacted in a 320 px box Fourier cropped 4x to 80 px box. The initial model was generated in cryoSPARC™, performing the initial pre-processing steps described above independently to the Relion workflow. Following one round of 2D and 3D classification (the latter with C1 symmetry), the best 3D class, displaying clear secondary structure features and consisting of 307,098 particles, was chosen. Refinement using C7 symmetry and solvent flattening resulted in a 3.4 Å reconstruction. This stack of particles was subjected to CTF refinements, Bayesian polishing and further two 3D classification rounds without particle alignment, after which the best 268,823 particles were selected. Subsequent refinement, masking and sharpening yielded the final 3.1 Å map. The map resolution was determined based on the gold-standard 0.143 criterion.

### Model building, validation, and structural analysis

AlphaFold structure prediction of Mus musculus Pannexin-1 (AF-Q9JIP4-F1-model_v2.pdb) was used as a starting model^28,29^. Model re-building was performed in Coot^30^ and ISOLDE^31^. The final model was refined with anisotropic atomic B-factors in reciprocal space using REFMAC^32^. Molprobity score, clash score, rotamer and Ramachandran analyses were performed using MOLPROBITY^33^. Local resolution was estimated in cryoSPARC. 3D Fourier shell correlation was calculated using the remote 3DFSC Processing server (https://3dfsc.salk.edu/)^14^.

## Supplementary Information

Supplementary Information.

## Data Availability

Atomic coordinates for the mPANX1 have been deposited in the Protein Data Bank with accession code 8A3B, and the cryo-EM density map has been deposited in the Electron Microscopy Data Bank with the accession code EMD-15110.
