# Supplementary material for: Direct cell extraction of membrane proteins for structure–function analysis
Source: Sci Rep. 2023 Jan 25;13:1420. doi: 10.1038/s41598-023-28455-w (PMC9876986; doi:10.1038/s41598-023-28455-w)
Supplement: Supplementary file 1 — Supplementary Information. [file 41598_2023_28455_MOESM1_ESM.pdf]

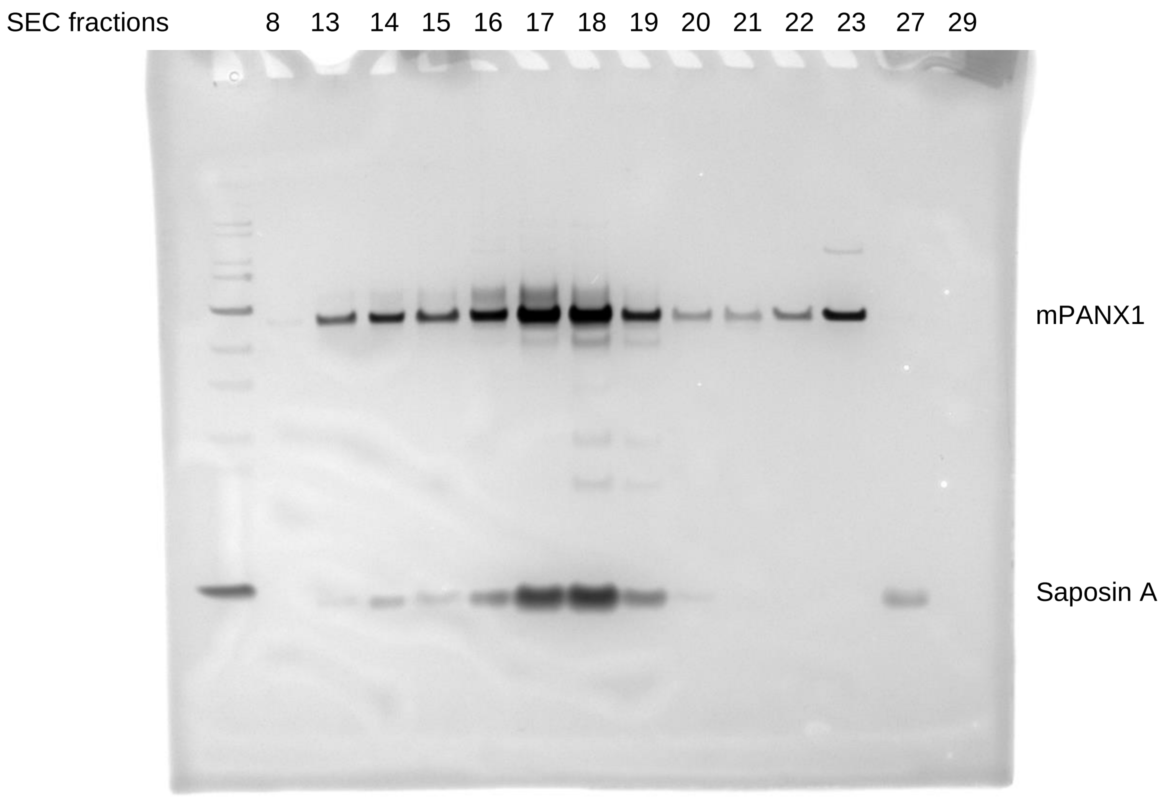

Supplementary figure 1. Shows the unprocessed and uncropped SDS-PAGE image presented in Figure 1E.

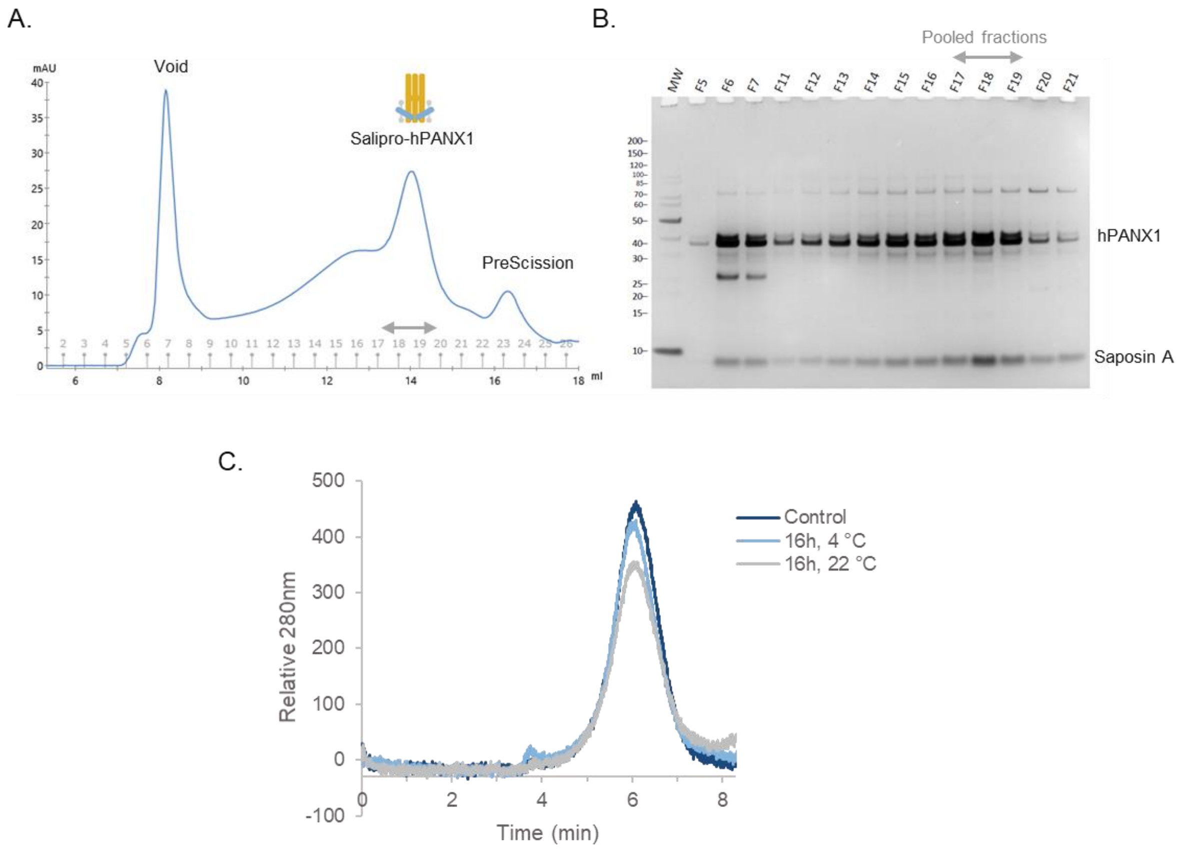

Supplementary figure 2. Direct cell extraction of Salipro-hPANX1. The hPANX1-GFP fusion protein was designed to contain c-terminal ^{10}His/EPEA affinity tags with a PreScission protease cleavage site upstream of GFP (hPANX1-PreScission-GFP-^{10}His/EPEA). Protein purification was achieved using C-tag affinity purification resin binding to the EPEA tag, followed by on-column PreScission cleavage. (A) Eluted Salipro-hPANX1 particles were concentrated by ultrafiltration and further purified by preparative SEC. (B) Collected SEC fractions were analysed by reducing SDS-PAGE using protein silver staining confirming the sole presence of hPANX1 and saposin A making up the Salipro-hPANX1 nanoparticles. SEC peak fractions 17-20 were pooled, concentrated and flash frozen in liquid nitrogen for -80 °C storage. (C) To evaluate Salipro-hPANX1 stability, particles were thawed and incubated for either 16h at 4 °C or at 22 °C followed by analytic SEC. The control sample was analyzed immediately after freeze-thawing without any further incubation.

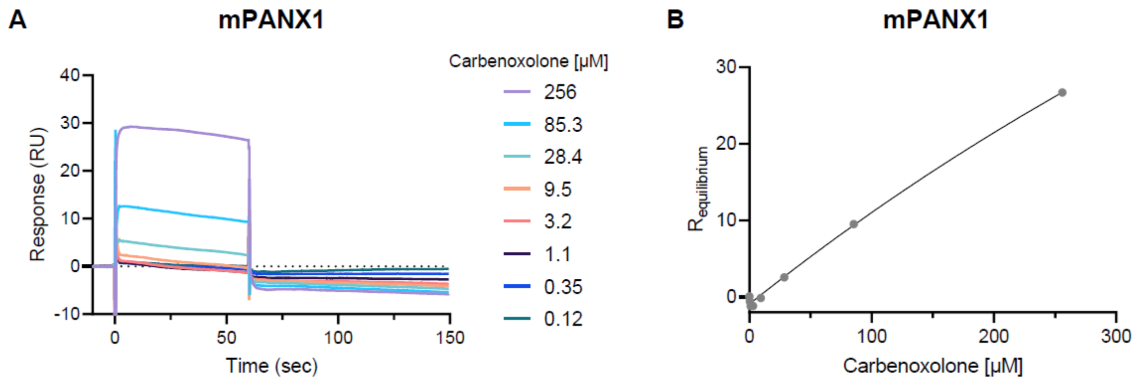

Supplementary figure 3. SPR analysis of His-Salipro-mPANX1 particle binding to carbenoxolone (A) Concentration series of carbenoxolone was injected over a His-Salipro-mPANX1 coated surface for 60 seconds followed by a 90 second dissociation time. (B) The equilibrium curve does not have sufficient curvature to accurately determine the KD. Data is representative of n=3.

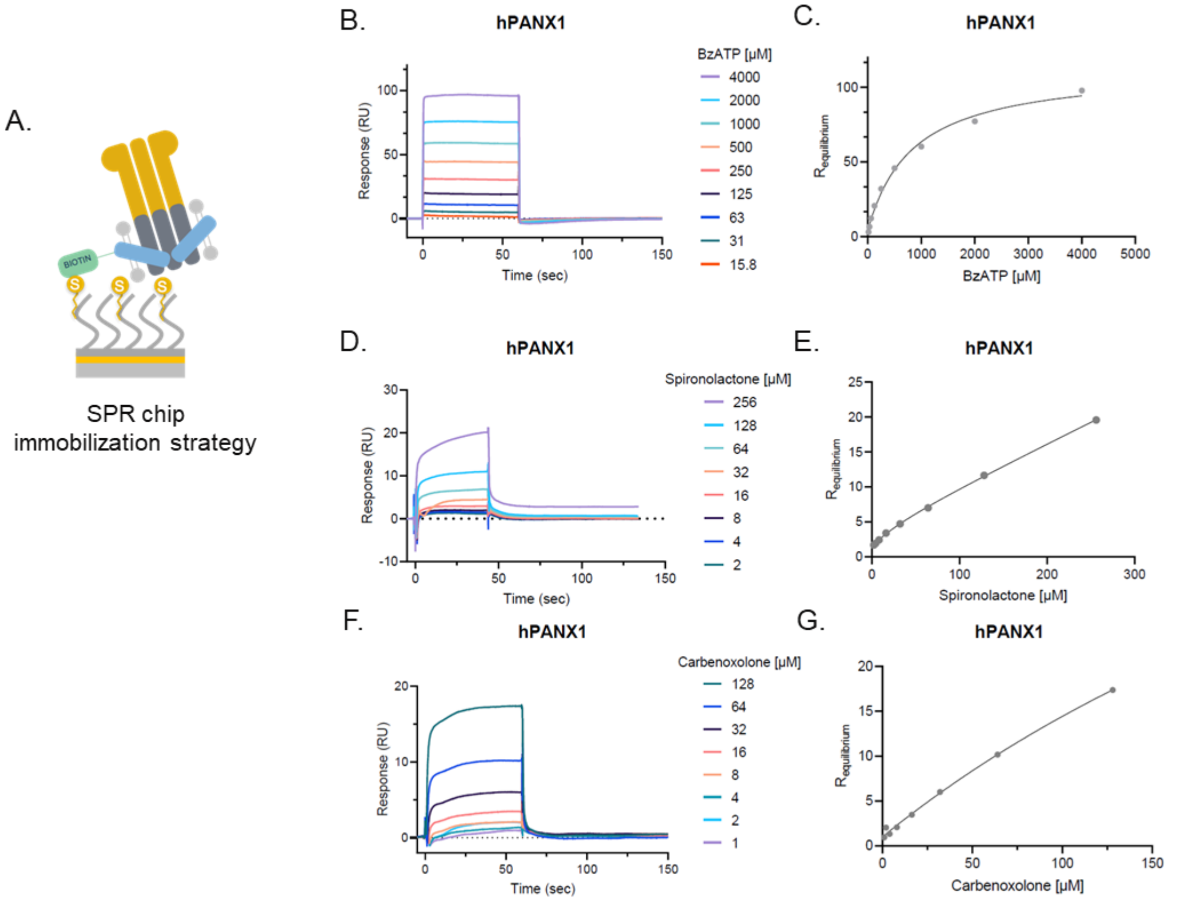

Supplementary figure 4. SPR analysis using biotin-Salipro-hPANX1 particle (A) Schematic illustration of biotin-Salipro-hPANX1 nanoparticles immobilization to a sensor chip coated with streptavidin (S). (B) Concentration series of BzATP was injected over a human PANX1 coated surface for 60 seconds followed by a 90 second dissociation time. (C) Equilibrium analysis showed a KD of 837µM ± 140µM. (D) Concentration series of spironolactone was injected over the surface for 45 seconds followed by a 90 second dissociation time. (E) The equilibrium curve does not have sufficient curvature to accurately determine the KD. (F) Concentration series of carbenoxolone was injected over a hPANX1 coated surface for 60 seconds followed by a 90 second dissociation time. (G) The equilibrium curve does not have sufficient curvature to accurately determine the KD. All data is representative of n=3**.**

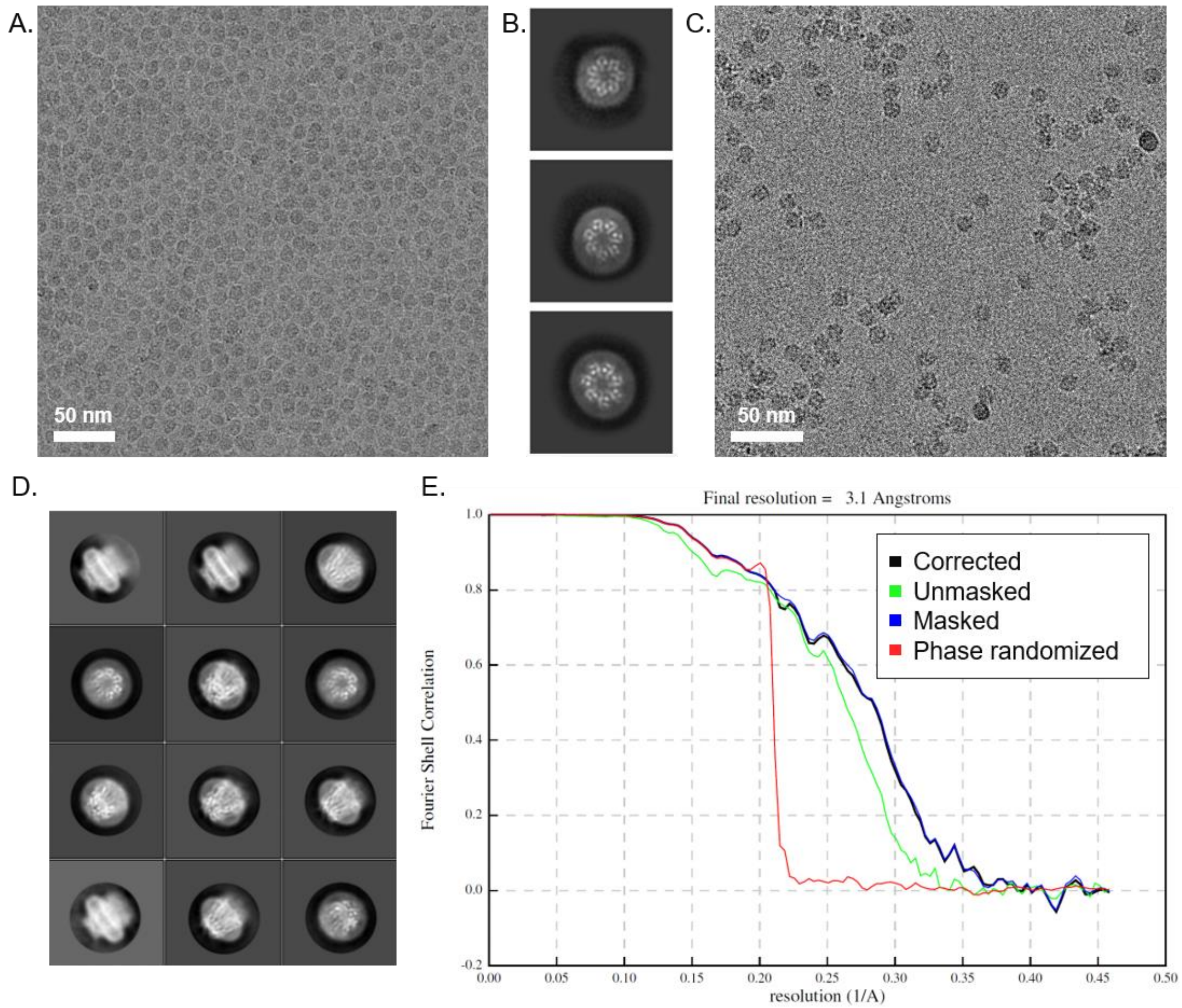

Supplementary figure 5 - Representative micrographs, 2D class averages and Fourier shell correlation curve for the Salipro-mPANX1 datasets. (A) Representative cryo-EM micrograph and (B) 2D class averages for the dataset without fluorinated Fos-Choline 8. (C) Representative cryo-EM micrograph and (D) 2D class averages for the dataset with 0.5 mM fluorinated Fos-Choline 8. (E) Fourier shell correlation (FSC) curve plot.

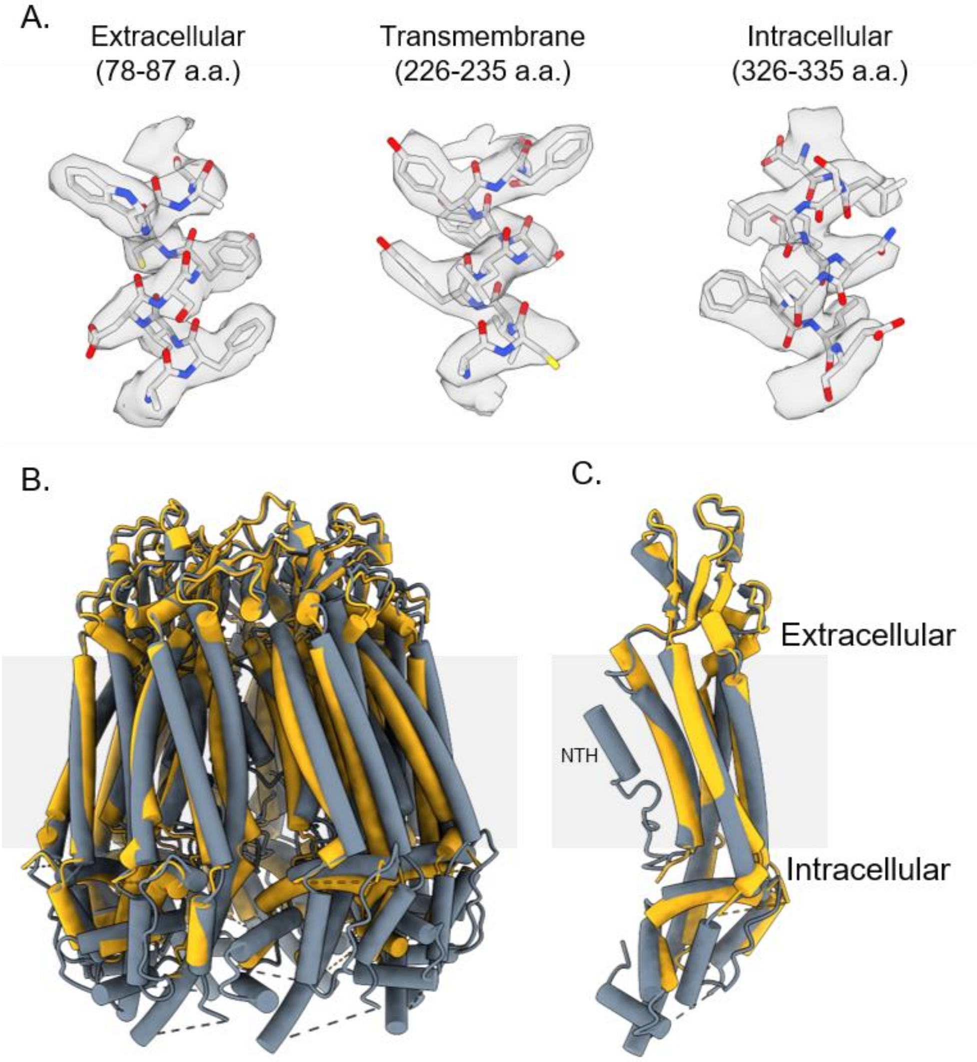

Supplementary figure 6 – Representative density and comparison to hPANX1 (PDB code 6WBF). (A) Representative density from extracellular, transmembrane and intracellular regions. (B) and (C) Comparison of Salipro-mPANX1 (shown in yellow) heptamer (B) and protomer (C) to hPANX1 (shown in grey). The intra-membrane N-terminal domain (NTH) resolved in the hPANX1 structure, but not in Salipro-mPANX1, is highlighted.

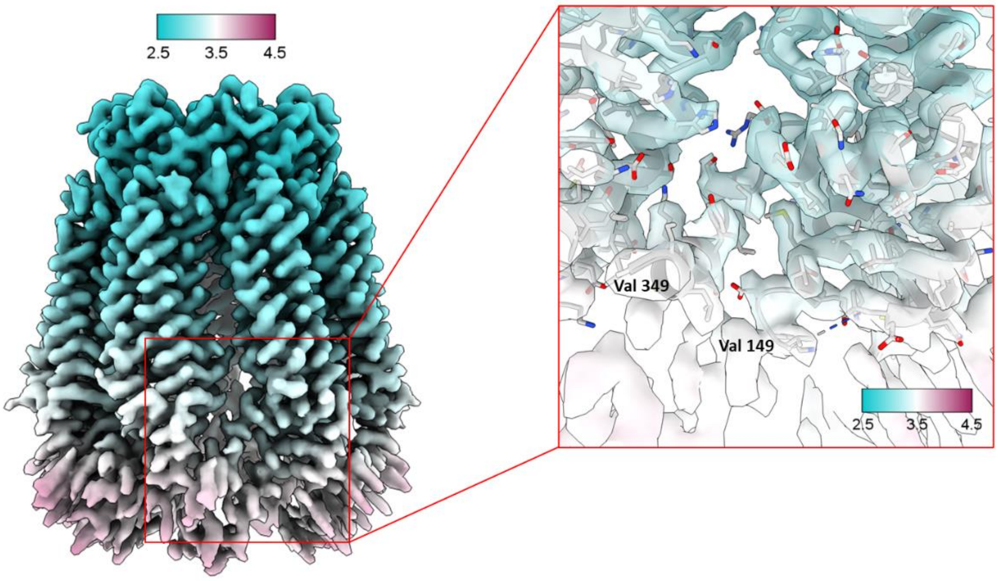

Supplementary figure 7. Cryo-EM map of Salipro-mPANX1 coloured according to local resolution. The inset displays the zoomed-in intracellular region with the last modelled residues labelled.

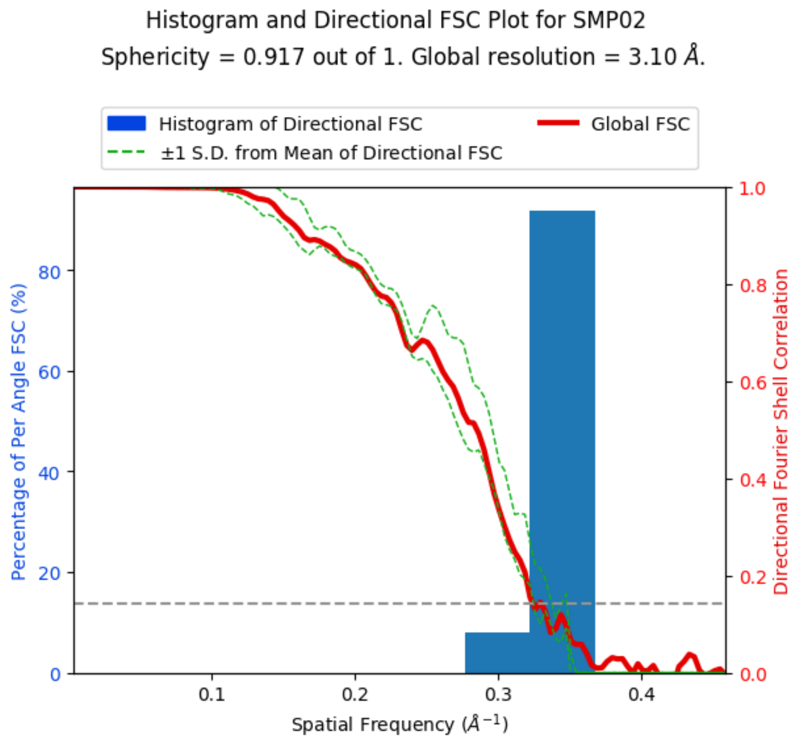

Supplementary figure 8. Graph showing the spread of 3D FSC values overlaid on the global half-map FSC curve.

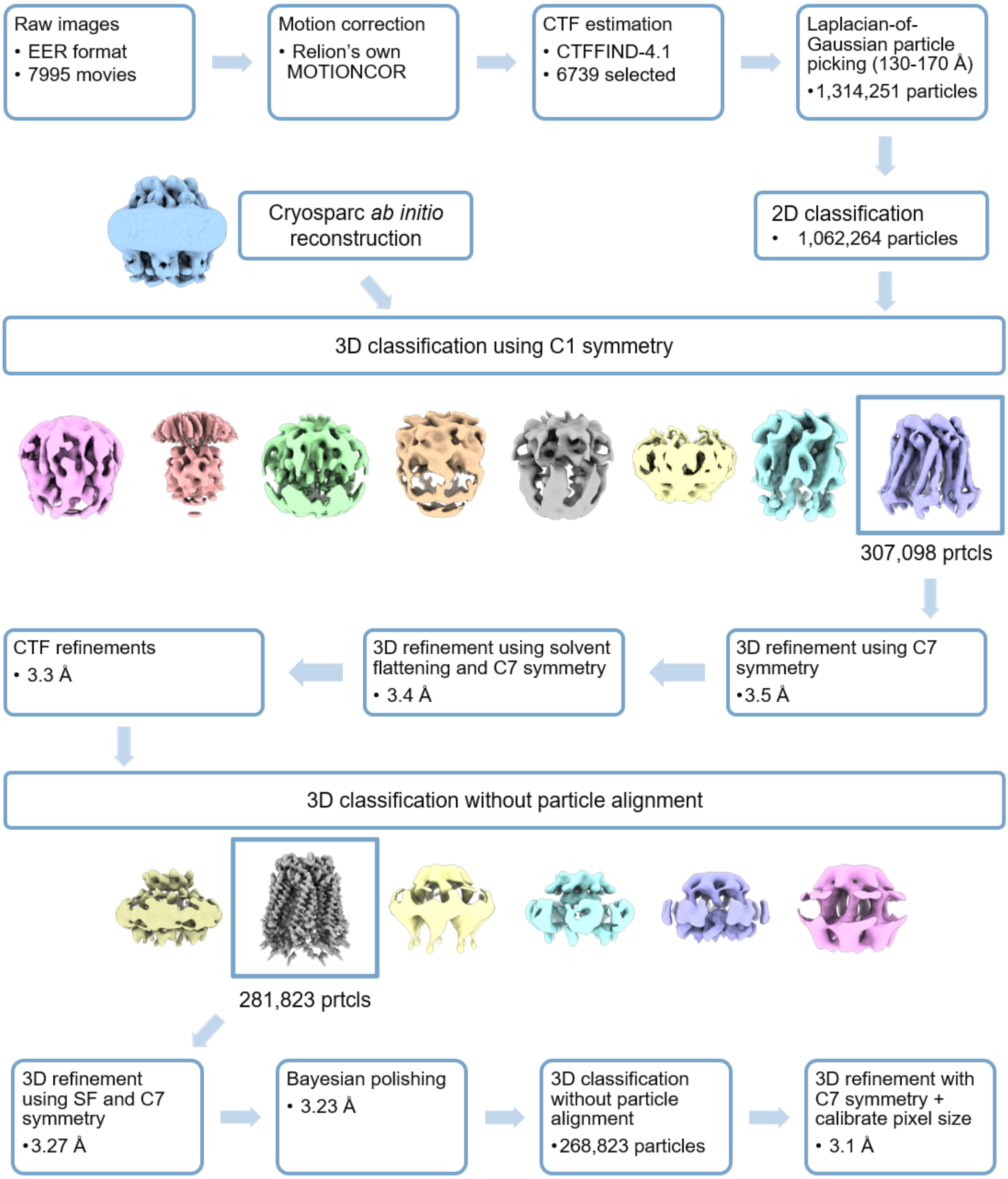

Supplementary figure 9. Cryo-EM image processing workflow. A detailed description of the data-analysis pipeline can be found in the Methods.

	mPANX1 (µM)	hPANX1 (µM)
BzATP	720 ± 133	837 ± 140
Carbenoxolone	ND	ND
Spironolactone	160 ± 10	ND

Supplementary Table 1: Summary of KD values determined from SPR experiments described in Figure 2B-C and Supplementary Figure 3.

	#1 Salipro-mPANX1 (EMDB-15110) (PDB 8A3B)
**Data collection and processing**	
Magnification	165,000x
Voltage (kV)	300
Electron exposure (e–/Å^2)	40.24
Defocus range (μm)	-0.5 to -2
Pixel size (Å)	0.727
Symmetry imposed	C7
Initial particle images (no.)	1,314,251
Final particle images (no.)	268,823
Map resolution (Å)	3.1
FSC threshold	0.143
Map resolution range (Å)	2.52-5.47
**Refinement**	
Initial model used (PDB code)	AF-Q9JIP4-F1-model_v2
Model resolution (Å)	n/a
FSC threshold	n/a
Model resolution range (Å)	n/a
Map sharpening *B* factor (Å^2)	-136.316
Model composition	
Non-hydrogen atoms	14448
Protein residues	1792
Ligands	n/a
B factors (Å^2)	
Protein	31.948
Ligand	n/a
R.m.s. deviations	
Bond lengths (Å)	0.015
Bond angles (°)	1.507

Validation	
MolProbity score	1.36
Clashscore	6.56
Poor rotamers (%)	0
Ramachandran plot	
Favored (%)	97.51
Allowed (%)	2.49
Disallowed (%)	0

Supplementary Table 2. Cryo-EM data collection, refinement, and validation statistics
